# Integrity of Organic Foods and Their Suppliers: Fraud Vulnerability Across Chains

**DOI:** 10.3390/foods9020188

**Published:** 2020-02-14

**Authors:** Saskia M. van Ruth, Leontien de Pagter-de Witte

**Affiliations:** 1 Food Quality and Design, Wageningen University and Research, P.O. Box 17, 6700 AA Wageningen, The Netherlands; 2 Wageningen Food Safety Research, part of Wageningen University and Research, P.O. Box 230, 6700 AE Wageningen, The Netherlands; leontien.depagter@wur.nl; 3 Institute for Global Food Security, School of Biological Sciences, Queen’s University, 19 Chlorine Gardens Belfast BT9 5DL, UK

**Keywords:** banana, egg, fraud, olive oil, organic, mitigation, pork, vulnerability

## Abstract

Organic foods are frequently targeted by fraudsters. Examination of underlying factors helps to reduce fraud vulnerability and to prevent fraud. In this study, the fraud vulnerability of five actors from each of four chains were examined using the SSAFE food fraud vulnerability assessment tool: the organic banana, egg, olive oil and pork supply chains. The organic chains appeared slightly less vulnerable than conventional chains due to fewer opportunities for fraud and the more adequate controls being present. On the other hand, organic chains were associated with enhanced vulnerability resulting from cultural and behavioral drivers. Generally, actors in the organic olive oil and pork chains were more vulnerable than those from the banana and egg chains. However, high risk actors were not limited to particular chains. Across the whole group of actors in organic chains, three groups in terms of cultural/behavioral drivers were distinguished: a low vulnerability group, a group facing more external threats and a group presenting fraud vulnerability in general and in particular from within their own company. Ethical business culture and criminal history scores of businesses correlated significantly. This implies that the climate in a company is an important factor to consider when estimating the exposure of businesses to food fraud.

## 1. Introduction

Over the last decades the interest in organic food products has increased due to the perceived benefits in terms of nutrition, health and safety, quality, animal welfare, local production and environmental aspects [1]. Whether these perceptions are based on facts is the subject of many debates [2,3,4]. It is a fact, however, that the demand for organic food products is growing at a steady rate [5].

There are various standards and regulations for organic production available. Codex Alimentarius Guidelines [6] and the International Federation of Organic Agriculture Movements Basic Standards provide a minimum baseline for national and regional standards on organic production worldwide [7]. In addition, in the European Union, production and labelling of organic foods is governed by the Council Regulation (EC) no. 834/2007 [8]. Other areas and countries in the world have their own regulations.

Organic products retail at a higher price than their conventional counterparts. It is also very hard to distinguish organic from conventional produce visually, but even with analytical tests authentication is a challenge. These opportunities and drivers lead to some individuals being tempted to outsmart customers and replace organic products with conventional ones. The smarter fraudsters mix conventional produce with a small amount of certified organic produce, like in a recent case in the United States [9]. All organic producers are inspected by organic inspection bodies, which may be private or managed by governments. Although this certainly helps to improve transparency, it may not be sufficient in stopping illicit practice in its tracks. These illegal activities not only deceive consumers who pay for products they do not get, and pay for products they do not want, but they also harm organic farmers who are playing by the rules and cannot compete with the lower prices usually offered. It causes incalculable damage to the confidence consumers have in organic products.

Although frauds mixed with organic food products surface frequently, not all actors are similarly vulnerable to internal and external threats. Vulnerability depends on a number of factors. In earlier studies, we developed a conceptual and analytical framework based on the Routine Activities Theory for understanding food frauds and developing mechanisms to prevent and reduce fraud vulnerability [10]. For the purpose of prevention, more can be gained from concentrating on the opportunities and motivational drivers that increase the risk of fraudulent activities as well as mitigation measures that reduce this risk. Examination of the fraud vulnerability across actors and underlying factors provides insights into the weaker and the more resilient nodes in supply chains. The SSAFE food fraud vulnerability assessment (FFVA) tool was developed on the basis of this conceptual framework [11] and allows for a detailed analysis of the fraud vulnerability of actors in food supply chains.

Considering the attractiveness of the organic food supply chains, we investigated the fraud vulnerability of actors in the organic banana, egg, olive oil and pork supply chains in the current study using this SSAFE FFVA tool. We were particularly interested in the distinction of high risk actors from others and elucidation of the underlying fraud factors.

## 2. Materials and Methods

### 2.1. Respondents

European businesses in the middle of the organic banana, organic egg, organic olive oil and organic pork supply chains (processors, wholesale) were assessed for their fraud vulnerabilities. Primary producers or retailers were not included. In each supply chain, five actors were interviewed. The banana actor group consisted of three businesses that were located in the Netherlands, one in Germany and one in the United Kingdom. Two were large-sized businesses (>100 employees) and three were smaller sized (<100 employees). The egg group comprised four businesses from the Netherlands and one from Germany, and concerned three large-sized business and two smaller sized businesses. The olive oil group involved one business from each of the following EU member states: France, Greece, the Netherlands, Portugal and Spain. These were all smaller-sized businesses. Finally, all actors of the organic pork group were based in the Netherlands and were larger sized. In this article, the actors were coded as follows: organic banana: BAN; organic egg: EGG; organic olive oil: OO; organic pork: PORK. The commodity code was followed by a number for the particular actor. For instance, BAN1 refers to actor 1 of the banana supply chain.

### 2.2. FFVA

The FFVA consisted of 50 questions and associated three-level answering grids (low-medium-high vulnerability). Questions 6 and 7, which deal with counterfeiting, were excluded because they do not apply to the particular product groups. The answers associated with the three vulnerability levels are available in the tool documents [11]. Each business was interviewed according to the procedure described previously [12]. The selected answers were transformed to a score system. A score of 1, 2 and 3 was assigned to low, medium and high vulnerability answering options, respectively. This resulted in a matrix of 20 businesses x 48 questions. The matrix was subdivided according to the relationship of the questions with the three key elements (opportunities, motivations and control measures) and were similarly divided for the six fraud factor categories (technical opportunities, opportunities in time and place, economic drivers, behavioral drivers, technical controls and managerial controls) (Appendix A).

### 2.3. Data Analysis

Significant differences between the scores of actors in the four commodity supply chains were assessed by analysis of variance (ANOVA). This was applied to each FFVA question. To assess correlations between ethical business culture vulnerability levels and criminal offences, Spearman’s rank correlation tests were performed on all related scores and Spearman’s rho (r_s_) was calculated. Agglomerative hierarchical cluster (AHC) analysis was applied to examine similarities in scores patterns of individual actors.

The relative frequency of high vulnerability answers was calculated for each actor, for example, the number of high vulnerability answers of actor 1 in the banana supply chain was divided by 48 (total answers) and multiplied by 100%. The same calculations were conducted for each fraud factor category for each actor. Similarly, these calculations were carried out for each supply chain group across the five actors. For instance, the high vulnerability answers to the economic driver-related questions of the five banana actors were divided by the total number of answers (5 actors × 7 questions) and multiplied by 100%. The frequencies for the key elements (opportunities, motivations and controls) were calculated as the average frequencies of each of the two fraud factor categories. Thus, the weighted frequencies were balanced for the number of factors in each category. Significant differences between the frequencies of groups were assessed by analysis of variance (ANOVA). To explore patterns in these frequency data, principal component analysis (PCA) was carried out on the frequency data. XL stat (Addinsoft, New York, NY, USA) was used for all statistical analyses. A significance level of *p* < 0.05 was applied throughout the paper.

## 3. Results and Discussion

### 3.1. Vulnerability Differences Among Organic Chains and Comparison with Conventional Chains

The results of the assessments of the 20 actors in the organic banana, egg, olive oil and pork supply chains were collated and the frequencies of high risk answers were calculated.

**Figure 1:**
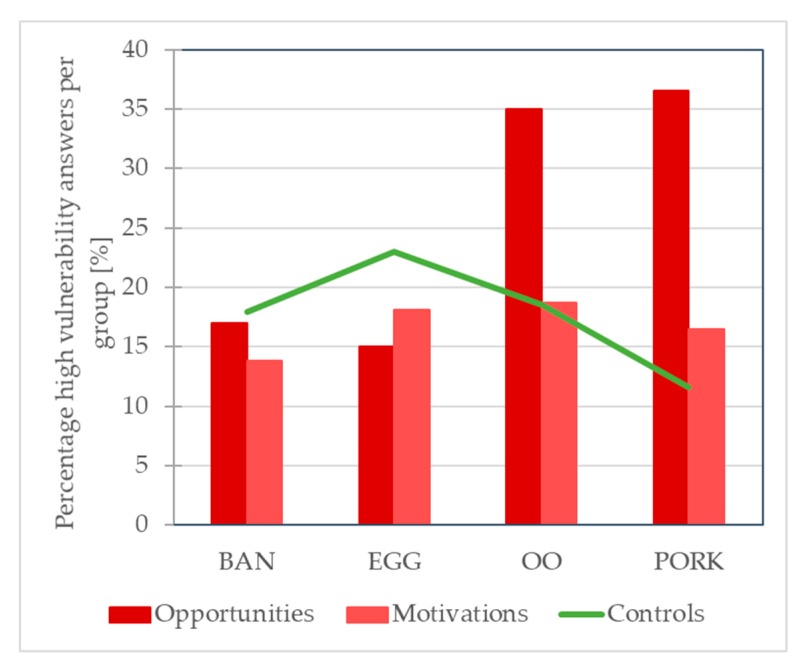
Relative frequencies of high vulnerability answers to the questions associated with the three key elements (opportunities, motivations, controls) provided by the banana (BAN), egg (EGG), olive oil (OO) and pork (PORK) supply chain actors.

The percentages of high risk answers for the key elements opportunities, motivations and controls are presented for the four supply chains in Figure 1. It shows that, overall, the organic olive oil and pork supply appear more vulnerable compared to the banana and egg supply chains due to enhanced opportunities (cumulated scores). Vulnerability seems food supply chain-related to some extent, which was observed for conventional food supply chains also; however, for those chains, differences between the chains were more distinct [13]. Because all businesses operated in the middle of the chain, we cannot draw any conclusions regarding differences between tier groups.

The higher frequencies for olive oil and pork supply chains appeared fairly consistent within the pork supply chain group. However, for the olive oil chain, large variation in the group was observed (Table 1). Although the motivations appeared to differ less than the opportunities, high risk motivation-related answer frequencies differed significantly across groups (ANOVA, *p* < 0.05).

**Figure 2:**
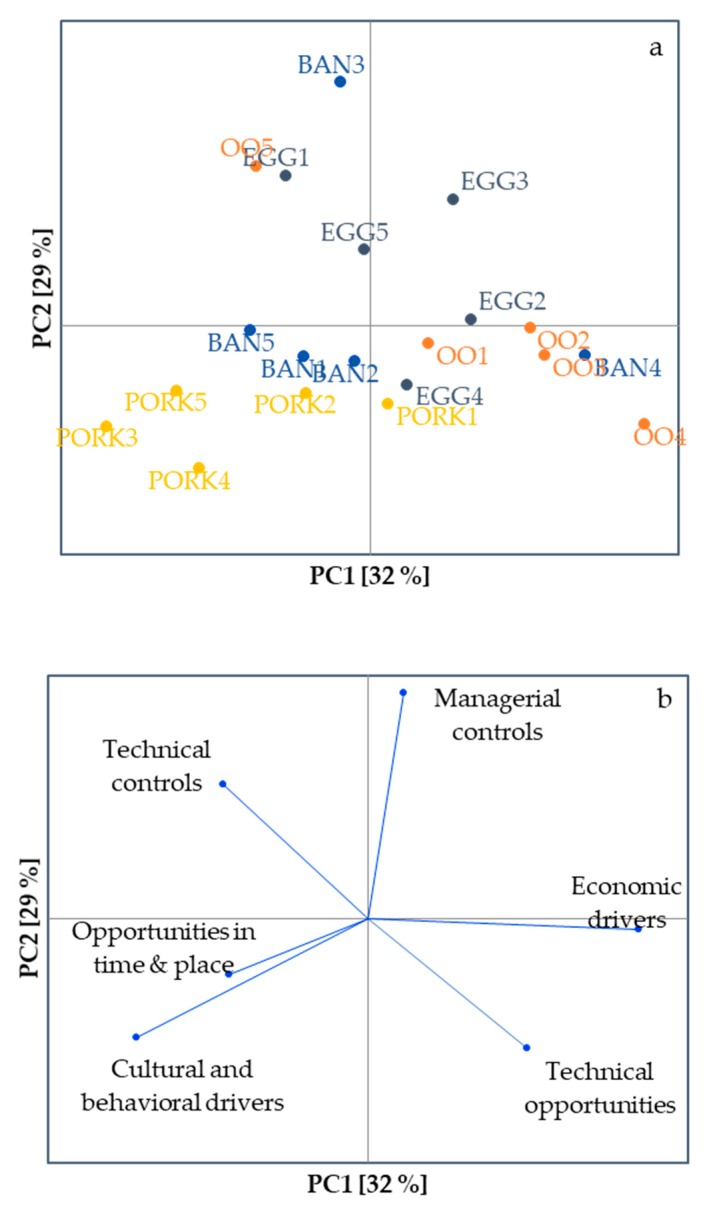
First two dimensions of a principal component analysis on the high risk answer frequencies of the six fraud factor categories provided by the banana (BAN), egg (EGG), olive oil (OO) and pork (PORK) supply chain actors: (**a**) scores plot; (**b**) loadings plot. Actors are indicated by their supply chain code followed by a number (e.g., BAN1).

Patterns in high risk frequencies of the questions associated with the six fraud factor categories across the various actors were explored by PCA (Figure 2). The five actors in the pork and three actors in the banana supply chains presented high vulnerability resulting from opportunities in time and place as well as cultural and behavioral drivers. Four out of the five actors in the olive oil supply chain showed high vulnerability scores in terms of economic drivers and technical opportunities. Compared with previous work on conventional supply chain actors, however, actors showed a more dispersed pattern according to type of supply chain [13]. In the latter study, differences between conventional supply chains indicated that the spice supply chain presented the most vulnerability challenges resulting from opportunities, motivations and lack of controls and was clearly distinguished from the other chains. The less distinct pattern may be due to the selection of chains, that is, in the current study, organic banana, egg, olive oil and pork were examined, and previously conventional fish, meat, milk, organic banana and spices were targeted. It is also feasible that the actors in the current supply chains are less homogeneous in vulnerability due to their organic production system, which adds another vulnerability dimension. It is remarkable that the high risk answer frequencies of the opportunities were lower for the organic supply chains (26% for the organic banana, egg, olive oil and pork supply chains) than for the conventional supply chains (35% on average for the fish, milk, meat and olive oil supply chains). Similarly, the high risk answer frequency was also lower for the organic chains (18%) for the controls compared to the conventional chains (30%). For the motivations, a slightly higher high-vulnerability answer frequency was observed for the organic chains, that is, 17% versus 14%. The results of the conventional spice chain was left out in this comparison, because of their exceptionally high vulnerability and a lacking organic counterpart chain. The differences above imply a somewhat lower vulnerability of the organic chains due to reduced opportunities and enhanced controls. Although the chains examined in both studies are similar and have a comparable fraud occurrence [14], it needs to be kept in mind that the comparison is not made with identical chains.

Examining individual fraud factors, calculations revealed significant differences between the four organic supply chains for 23 fraud factors, that is, three opportunity-related factors, 14 motivations-related factors and six control measures (ANOVA, *p* < 0.05). Surprisingly, the number of factors differing significantly was exactly the same as in the previous study in which supply chains of conventional production management were evaluated [13]. However, the proportions of factors associated with the key elements differed. Previously, 4 opportunity-related factors, 9 motivation-related factors and 10 control measures presented significant differences between tiers. It is remarkable that for both the organic and conventional chains, primarily differences in opportunities in time and place, as well as in cultural and behavioral drivers were observed in addition to differences in controls. It appears that actors see fairly consistent technical opportunities and economic drivers to adulterate their product across chains. This is an interesting phenomenon—any product appears to exhibit traits that can be manipulated for illicit gain according to their supply chain actors. Long, misty supply chains will favor fraud, but so will a poor morale. Because a supply chain is usually determined by the type of product, that is, some products simply have a long supply chain, cultural and behavioral drivers are the components that vary most from company to company. Therefore, examined fraud factors associated with these drivers in greater detail, which is discussed in the next section.

### 3.2. Vulnerability Differences Among Organic Chain Actors Due to Cultural and Behavioral Drivers

**Figure 3:**
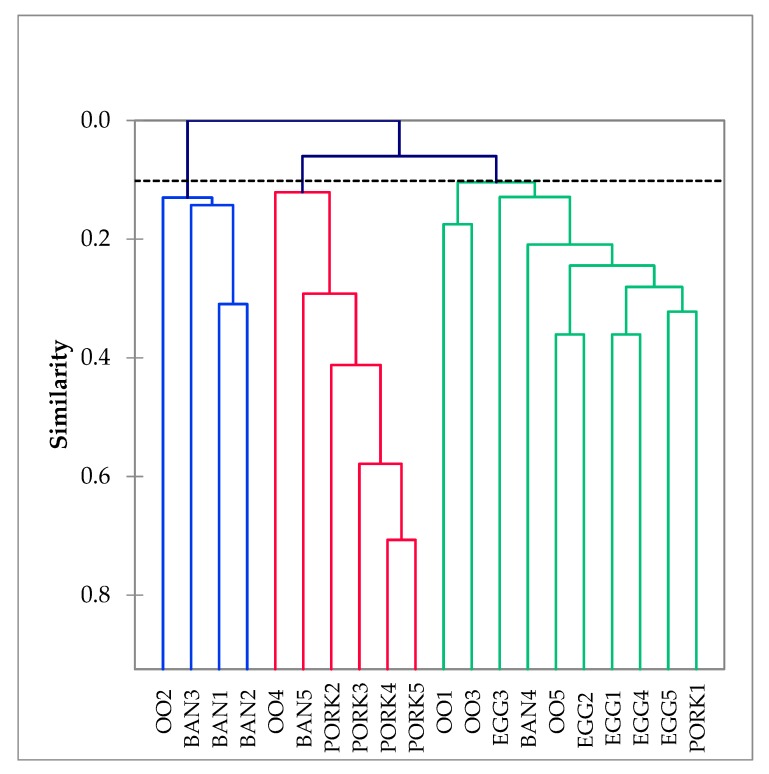
Dendrogram of the agglomerative hierarchical cluster analysis on the scores for the cultural and behavioral driver factors provided by the banana (BAN), egg (EGG), olive oil (OO) and pork (PORK) supply chain actors. Actors are indicated by their supply chain code followed by a number (e.g., BAN1).

Thus, culture and behavior appear as fairly discriminatory factors for their fraud vulnerability. Therefore, the similarities of actors in vulnerabilities resulting from cultural and behavioral drivers, independent of supply chain, were examined. AHC analysis of the scores of the actors for the cultural and behavioral driver questions revealed three groups of actors (Figure 3).

The first group presented a generally low vulnerability, except for the corruption levels of the countries in which the own company and that of the suppliers operate. This group included three banana actors and one olive oil actor. A high vulnerability group was also distinguished and characterized by the factors “own company’s criminal offences”, “own ethical business culture” and “own financial strains imposed on suppliers”, as well as “the ethical business culture of the suppliers” and “the ethical culture in the branch of industry”. This group concerned four of the five actors in the organic pork industry, as well as one banana and one olive oil supply chain actor. This group possessed traits that are worrisome when it comes to potential fraud in general, along with fraud from within the own company in particular. A second vulnerable group was associated with high scores for “criminal offences of the suppliers” and “criminal offences of the customers”, as well as “victimization”. This group seemed particularly vulnerable to becoming a victim of external fraud according to these factors. Although actors from some supply chains (pork, banana) were more represented in a particular group, it was also obvious that high- and low-risk actors with regard to cultural and behavioral drivers were found across supply chains. Although high-risk actors may have been more prevalent in one chain (e.g., pork), they were found in other chains too. With this in mind, it was not surprising that fraud surfaces in any food supply chain, from cheap commodity products to expensive specialty foods.

Food fraud is predominantly an occupational fraud, a white collar crime. Company climate (business culture) is, therefore, very important in this respect. Ethical business culture can be a proxy measure of fraud and is negatively associated with the pressure in a company and strength of rationalization [15,16]. Murphy and Free [15] reported the importance of a business culture in which employees make decisions in their own or the organization’s best interests without being bothered by ethical concerns when fraud is perpetrated. This type of business culture is associated with motives such as a malevolent work environment and social incentives and pressures, as well as rationalizations that are primarily oriented toward others. One specific rationalization—“helping the company”—draws attention to the phenomenon of unethical pro-organizational behavior. Frauds do not necessarily need to result in direct individual gain but may ‘just’ be in the interest of the company. In order to combat this kind of unethical activities, it is key to maintaining an appropriate ethical corporate business culture. Reducing occupational fraud can be best achieved through enhancing the ethical corporate culture according to Suh and co-workers [17]. They presented two mediating variables, ethical culture and monitoring control. However, only an improved ethical culture was significantly negatively related to the occupational fraud frequency. These findings imply that investing in ethical culture is crucial in preventing occupational fraud, and reversed, a weak ethical culture creates a climate in which illicit activities flourish. To favor a good ethical business culture, three essential elements should be present: (1) the existence of core ethical values embedded throughout the business; (2) the establishment of a formal ethics program including ethics training; (3) the continuous presence of ethical leadership, this being an appropriate “tone at the top”, as reflected by the management board of the business [18]. Considering the importance of ethical business culture as a determinant of the level of food fraud vulnerability, these elements deserve attention in any mitigation plan.

Thus, considering the clusters of companies, vulnerability and the studies above, ethical business culture appears fairly important. The FFVA included questions on the ethical business culture of the own company, the supplier and the branch of industry (Table 2). Similarly, questions on criminal offences/historical evidence of fraud in the branch provided information on occurrence of offences at the level of the own company, the supplier and the branch of industry (Table 2). It is interesting to note that the own company was always rated lowest, followed by the supplier and finally by the branch of industry. This phenomenon is in agreement with the “alien conspiracy theory”, which suggest that outsiders and outside influences are to blame for the prevalence of crime [19]. Correlation between the two aspects mentioned above provides information on the relationship between ethical business culture and criminal behavior in the food supply chains. The ethical business culture vulnerability levels and the assigned levels to the offence rates revealed a significant correlation (r_s_ = 0.376; *p* = 0.003). Hence, ethical business culture is a very important aspect to consider when mitigating food fraud. Whether the food fraud is pre-planned or not, is a one-off incident, is a series of incidents, is embedded and routine practice, is for personal gain or is to “help the company”, the fraudulent behaviors are influenced by the “company climate” components irrespective of chain, tier and scale of the company. A key issue in food frauds is that such behaviors may become normalized across food chains and branch of industries. This culturally embedded behavior perpetuates unethical practice. It also works contagiously, as businesses that may be disadvantaged through such practices are forced to “swim with the tide”, eventually resulting in an “unethical slippery slope” [20], with some sectors becoming more contaminated than others.

## 4. Conclusions

Organic chains seem somewhat less vulnerable to fraud than their conventional counterparts due to fewer opportunities and more adequate controls, but on the other hand, enhanced motivational drivers add to vulnerability. Fraud awareness and guidance are also better developed. Opportunities in time and place, as well as cultural and behavioral drivers, seem the main causes of differences between chains, both for actors in organic and conventional management systems. Across the whole group of actors in organic chains, three groups in terms of culture/behavior can be distinguished: a low vulnerability group, a group being particularly threatened externally and at increased risk to become a victim and a group that is generally vulnerable and particularly vulnerable to threats from within their own company. High risk is present across chains, and is not limited to particular chains. Ethical business culture, the climate in a company, appears to be a very important factor of food fraud vulnerability and requires sufficient attention in fraud mitigation plans.

## Figures and Tables

**Table 1:** Relative frequencies of high vulnerability answers to questions associated with the three key elements and provided by the individual supply chain actors: opportunities, motivations and controls. The occurrence of high vulnerability answers is indicated by the red color intensity of the cells.

Actor	Opportunities	Motivations	Controls
BAN1	10	4	5
BAN2	20	4	6
BAN3	0	7	49
BAN4	30	32	13
BAN5	25	22	17
EGG1	0	11	37
EGG2	20	29	19
EGG3	23	21	34
EGG4	33	18	6
EGG5	0	11	19
OO1	10	18	6
OO2	30	14	13
OO3	63	21	22
OO4	40	29	0
OO5	33	11	52
PORK1	43	18	9
PORK2	53	15	20
PORK3	45	12	14
PORK4	33	23	5
PORK5	10	15	11

^1^ Supply chain in which the actor operates, followed by the actor number: BAN = organic bananas, EGG = organic eggs, OO = organic olive oil, PORK = organic pork.

**Table 2:** Scores of actors related to six questions of the food fraud vulnerability assessment regarding ethical business culture and criminal history of the own company, supplier and branch of industry ^1^.

Actor.	Ethical Business Culture Own Company	Ethical Business Culture Supplier	Ethical Business Culture Branch of Industry	Criminal Offences Own Company	Criminal Offences Supplier	Historical Evidence Branch of Industry
BAN1	1	1	1	1	1	1
BAN2	1	1	1	1	1	1
BAN3	1	1	1	1	1	1
BAN4	1	1	1	1	1	3
BAN5	1	2	2	1	2	3
EGG1	1	2	3	1	2	2
EGG2	1	1	2	1	1	1
EGG3	1	2	2	1	2	2
EGG4	1	1	1	1	1	2
EGG5	1	2	3	1	1	2
OO1	1	1	2	1	1	3
OO2	1	2	3	2	1	3
OO3	1	2	1	2	1	2
OO4	1	2	2	1	1	3
OO5	1	1	2	2	1	3
PORK1	1	1	2	1	1	3
PORK2	1	1	1	1	1	3
PORK3	1	1	1	1	3	3
PORK4	1	1	1	1	3	1
PORK5	1	1	1	1	3	3

^1^ Scores indicate vulnerability levels of answers provided by the actors: 1 = low, 2 = medium, 3 = high. Actors are indicated by their supply chain code followed by a number (e.g., BAN1): BAN = organic bananas, EGG = organic eggs, OO = organic olive oil, PORK = organic pork.

## Appendix A

**Table A1:** Key elements, fraud factor categories and their associated fraud factors from the Food Fraud Vulnerability Assessment.

**Opportunities**	
**Technical Opportunities**	**Opportunities in Time and Place**
Q1. Complexity of adulteration raw materials	Q8. Access to production lines/processing activities
Q2. Availability of technology and knowledge to adulterate raw materials	Q9. Transparency in the chain network
Q3. Fraud detectability in raw materials	Q10. Historical evidence of fraud in raw materials
Q4. Availability of technology and knowledge to adulterate final products	Q11. Historical evidence of fraud in final products
Q5. Fraud detectability in final products	
**Motivations**	
**Economic Drivers**	**Cultural and Behavioral Drivers**
Q12. Supply and pricing raw materials	Q15. Organizational strategy of own company
Q13. Valuable components or attributes	Q16. Ethical business culture of own company
Q14. Economic health of own company	Q17. Criminal offences of own company
Q20. Economic health of supplier	Q18. Corruption level country of own company
Q26. Economic health of sector	Q19. Financial strains imposed on supplier by own company
Q30. Level of competition branch of industry	Q21. Organizational strategy of supplier
Q31. Price asymmetries	Q22. Ethical business culture of supplier
	Q23. Criminal offences of supplier
	Q24. Victimization of supplier
	Q25. Corruption level country of suppliers
	Q27. Criminal offences of customer
	Q28. Ethical business culture of branch of industry
	Q29. Historical evidence in branch of industry
**Controls**	
**Technical Controls**	**Managerial Controls**
Q32. Fraud monitoring system for raw materials of own company	Q38. Integrity screening of own employees
Q33. Verification of fraud monitoring system for raw materials of own company	Q39. Ethical code of conduct of own company
Q34. Fraud monitoring system for final products of own company	Q40. Whistle-blowing facilities of own company
Q35. Verification of fraud monitoring system for final products of own company	Q41. Contractual requirements of supplier
Q36. Information system of own company	Q45. Social control in chain network
Q37. Tracking and tracing system of own company	Q47. National food policy
Q42. Fraud monitoring system of supplier	Q48. Law enforcement in local chain
Q43. Information system of supplier	Q49. Law enforcement in chain network
Q44. Tracking and tracing system of supplier	
Q46. Fraud control in sector	
Q50. Contingency plan	
